# Artificial intelligence-based risk assessment tools for sexual, reproductive and mental health: a systematic review

**DOI:** 10.1186/s12911-025-02864-5

**Published:** 2025-03-17

**Authors:** Shifat Islam, Rifat Shahriyar, Abhishek Agarwala, Marzia Zaman, Shamim Ahamed, Rifat Rahman, Moinul H. Chowdhury, Farhana Sarker, Khondaker A. Mamun

**Affiliations:** 1 Department of Computer Science and Engineering, Bangladesh University of Engineering and Technology, Dhaka, 1000 Bangladesh; 2 AIMS Lab, Institute of Research, Innovation, Incubation and Commercialization (IRIIC), United International University, Dhaka, 1212 Bangladesh; 3 CMED Health Limited, Dhaka, 1206 Bangladesh; 4 Center for Computational and Data Sciences, Independent University Bangladesh, Dhaka, 1229 Bangladesh; 5 Department of Computer Science and Engineering, Independent University Bangladesh, Dhaka, 1229 Bangladesh; 6 Department of Computer Science and Engineering, United International University, Dhaka, 1212 Bangladesh

**Keywords:** Artificial intelligence (AI), Sexual, reproductive and mental health (SRMH), Triage, Symptom checker, Risk prediction

## Abstract

**Background:**

Artificial intelligence (AI), which emulates human intelligence through knowledge-based heuristics, has transformative impacts across various industries. In the global healthcare sector, there is a pressing need for advanced risk assessment tools due to the shortage of healthcare workers to manage the health needs of the growing population effectively. AI-based tools such as triage systems, symptom checkers, and risk prediction models are poised to democratize healthcare. This systematic review aims to comprehensively assess the current landscape of AI tools in healthcare and identify areas for future research, focusing particularly on sexual reproductive and mental health.

**Methods:**

Adhering to PRISMA guidelines, this review utilized data from seven databases: Science Direct, PubMed, SAGE, ACM Digital Library, Springer, IEEE Xplore, and Wiley. The selection process involved a rigorous screening of titles, abstracts, and full-text examinations of peer-reviewed articles published in English from 2018 to 2023. To ensure the quality of the studies, two independent reviewers applied the PROBAST and QUADAS-2 tools to evaluate the risk of bias in prognostic and diagnostic studies, respectively. Data extraction was also independently conducted.

**Results:**

Out of 1743 peer-reviewed articles screened, 63 articles (3.61%) met the inclusion criteria and were included in this study. These articles predominantly utilized clinical vignettes, demographic data, and medical data from online sources. Of the studies analyzed, 61.9% focused on sexual and reproductive health, while 38.1% addressed mental health assessment tools. The analysis revealed an increasing trend in research output over the review period and a notable disparity between developed and developing countries. The review highlighted that AI-based systems could outperform traditional clinical methods when implemented correctly.

**Conclusions:**

The findings indicate that integrating AI-based models into existing clinical systems can lead to substantial improvements in healthcare delivery and outcomes. However, future research should prioritize obtaining larger and more diverse datasets, including those from underrepresented populations, to reduce biases and disparities. Additionally, for AI-based healthcare interventions to be widely adopted, transparency and ethical considerations must be addressed, ensuring these technologies are used responsibly and effectively in practical scenarios.

**Supplementary Information:**

The online version contains supplementary material available at 10.1186/s12911-025-02864-5.

## Introduction

Sexually transmitted infections (STIs) and HIV are a big problem for global health [1, 2]. According to the World Health Organization (WHO), over a million people get an STI every day, and young people are most affected. Youth sexual and reproductive health (SRH) is a key part of the global health agenda, since half the world is under 25, with 1.8 billion people aged 10–24, 90% of whom live in low-to-middle income countries (LMICs) [3]. These numbers mean we need to get SRH sorted because neglect can have lifelong consequences [4].

Reproductive health in LMICs is also in crisis. Every year 16 million girls aged 15–19 give birth, that’s 11% of all global births, and 95% of those births happen in LMICs [5]. 220 million women in LMICs have unmet family planning needs [6]. Unwanted pregnancies and unsafe abortions are the result of this unmet need and often lead to pregnancy-related complications which are the leading cause of death for girls 15–19 in LMICs. Almost all the 3 million unsafe abortions happen within this age group [7].

Mental health (MH) is also a big problem for global health [8]. 70% of people globally don’t have access to good mental health care [9]. Severe mental illnesses like psychotic disorders are major contributors to years lived with disability. In LMICs access to mental health services is even more limited, 90% of people with schizophrenia don’t get the care they need [10]. The absence of specialized mental health professionals [11, 12] makes it worse, putting undue stress on caregivers [13] and increasing the risk of human rights violations against affected individuals. WHO predicts the shortage of healthcare professionals will rise from 7.2 million in 2013 to 12.9 million by 2035 [14]. We need to develop new tools and models to process large-scale, multi-domain data in real-time. We can use modern technology like artificial intelligence (AI) to improve risk forecasting, diagnosis and treatment.

Triage is a critical process in healthcare that prioritises patients based on the severity of their condition so that they get timely treatment, especially in emergency situations [15]. However traditional triage processes are not precise because of the limited availability of trained health workers. AI-based triage algorithms are a solution, they can analyse large data, identify patterns and allocate resources to high-risk patients [16, 17]. Similarly, AI-based symptom checkers are being developed to help users assess their health. These tools, often in the form of chatbots, help users assess symptoms, get a preliminary diagnosis and get guidance on the next steps [18]. These are very useful in LMICs where healthcare access is limited. Chatbot-based symptom checkers are a way for individuals to get early intervention and make informed health decisions. Risk prediction tools further enhance healthcare by analysing data from multiple sources such as electronic health records, demographic data and genetic information to generate personalized risk scores [19]. These are critical in supporting clinical decision-making, enabling healthcare workers to give personalized advice, optimize screening schedules and allocate resources better. By combining AI and machine learning, risk prediction models can detect new risk factors, predict adverse health outcomes and improve clinical accuracy.

The integration of SRH with (MH) in this review is because of the strong link between these two health areas. Adverse SRH outcomes like unintended pregnancies and STIs are linked to MH issues like anxiety and depression. Poor MH can also impact SRH behaviours like contraceptive use and healthcare seeking. Both SRH and MH have similar ethical and equity challenges, especially in LMICs where stigma, privacy, and consent are major barriers to healthcare. AI tools like triage systems, symptom checkers, and risk prediction models are cross-domain and can be used for self-assessment, urgent care prioritization, and early detection in both SRH and MH. Addressing these common challenges through integrated AI solutions can help in resource-poor settings.

Despite the growth of AI in healthcare, there is a gap in the development and application of AI-based risk assessment tools for SRH and MH. Traditional healthcare systems struggle to manage the complexity of these areas, especially in LMICs. Previous reviews have reviewed SRH and MH separately and not together, ignoring the common challenges and the potential for integrated AI solutions. While AI-based triage systems, symptom checkers and risk prediction models are being used, there is limited analysis of their cross-domain applicability, effectiveness, and ethical considerations.

This review aims to fill these gaps by synthesizing the existing literature on AI-based risk assessment tools in SRH and MH. It looks at the available AI solutions, identifies the challenges, and proposes strategies for developing and implementing better interventions. By addressing these gaps, this review aims to support the development of integrated, data-driven, and context-specific AI solutions that can inform decision-making and improve healthcare in LMICs where these innovations are most needed.

To the best of our knowledge, this is the first comprehensive review of AI-based risk assessment tools in SRH and MH. The review looks at key tools such as symptom checkers, triage systems and risk prediction models and highlights their potential in healthcare. It also evaluates existing AI algorithms, discusses solution effectiveness using specific metrics, and identifies the research gaps that need to be addressed to move forward with AI-based interventions in SRH and MH. The contribution of the review is to provide a holistic view of how AI can be used to improve healthcare and promote equity in resource-poor LMICs.

## Methods

We conducted a comprehensive search for research articles using various information sources. To ensure a comprehensive systematic review analysis, we conducted the PRISMA-P [20] methodology and assessed a total of 63 research articles. The pertinent articles were sourced from seven databases: ACM Digital Library, Science Direct, Sage, Springer, IEEE Xplore, Wiley, and PubMed.

### Eligibility criteria

The eligibility criteria play a crucial role in systematic reviews as they are shaped by the research questions that the paper aims to address. The specific inclusion and exclusion criteria are outlined below:

Inclusion Criteria (IC)

- IC1: Articles related to AI-based tools, including triage systems, symptom checkers, and risk prediction in SRMH, to focus on relevant AI applications in sexual, reproductive, and mental health.
- IC2: Peer-reviewed research articles to ensure high-quality, validated research.
- IC3: Articles involving human participants and relevant to healthcare emphasize real-world healthcare applications.
- IC4: Articles published between 2018 and 2023 to capture recent advancements in AI technology.
- IC5: Articles written in English to ensure accessibility for reviewers and readers.

Exclusion Criteria (EC)

- EC1: Articles unrelated to AI-based risk assessment tools in SRMH to maintain focus on relevant studies.
- EC2: Books, e-posters, review articles, scientific meeting papers, and non-research articles to prioritize primary research.
- EC3: Non-peer-reviewed articles to ensure research quality and reliability.
- EC4: Articles lacking sufficient data or proper methods to include only robust and informative studies.
- EC5: Articles proposing tools unrelated to human intervention studies to emphasize practical healthcare applications.
- EC6: Articles published before 2018 to exclude outdated technologies and findings.
- EC7: Articles not written in English to maintain consistency in comprehension and review.

### Search string

Our search string includes terms combined with Boolean operators (AND, OR) to indicate whether we want to include or exclude specific words. The resulting search sting:

(“Artificial intelligence” OR “Machine Learning” OR “Natural language Processing” OR “Large Language Model”) AND (“Triage” OR “Health Risk prediction” OR "Symptom checker") AND ("Sexual Health" OR "Reproductive Health" OR “Sexual and reproductive health” OR “Mental health”)

The advanced search string combinations for different databases (Additional file 1) were used to extract the relevant article.

### Search strategy

We developed our literature search strategy through a trial and error process refining a set of initial keywords for AI-based risk assessment tools in the SRMH domain by testing different Boolean combinations. This approach captured a broad range of relevant literature as the nature of interdisciplinary research at the intersection of technology and health is evolving. We chose databases with extensive coverage of medical and technological studies including PubMed, IEEE Xplore, ACM Digital Library, ScienceDirect, SAGE, Springer and Wiley. We selected these databases to retrieve a diverse range of high-quality interdisciplinary studies to analyse the potential and challenges of AI in health. The iterative nature of our search strategy development allowed us to refine continuously and exhaust the current landscape of AI in health solutions.

### Screening process

After the determination of the optimal search string, a Comma-Separated Value (CSV) file containing the search results was exported and obtained from each database. When a database did not support CSV output, the search results were exported in BibTeX format and then converted to CSV online using a conversion tool. Google Sheets was used as a collaborative spreadsheet application to help expedite and facilitate the screening process. In order to automatically detect and highlight duplicate entries based on their titles, conditional formatting was utilized together with specially constructed rules. The identified duplicate articles were then manually deleted.

We removed items that were irrelevant after doing an initial screening in which we evaluated the articles’ titles and abstracts to establish their relevancy. In order to improve the selection process and exclude articles that did not fulfil the eligibility requirements, the exclusion criteria were used during the second screening by reading the entire text. The articles that remained were selected for this systematic review.

We resolved disagreements between reviewers by consensus for inter-rater reliability. This decision was made to prioritize collaborative and nuanced assessments of relevance and quality of studies and to use the expertise of all authors. The consensus approach allowed for in-depth discussion and agreement on each paper to ensure rigorous and thorough screening in line with our systematic review objectives. This was particularly relevant given the interpretative nature of the criteria and the interdisciplinary nature of the research.

### Data collection process

Data was collected using a standardised form to ensure consistency throughout the review. The items in Table 1 were chosen based on widely used criteria in systematic reviews to define inclusion and exclusion criteria. These were tailored to the study’s scope and objectives and focused on key elements such as year, author, study design, AI tool(e.g. Triage, Symptom checker, Risk prediction), algorithm, metrics and outcomes that are commonly used in the literature. This structured approach allowed for the synthesis of data across different studies.

**Table 1:** Data items extracted from the selected research articles

Features	
Title of the research article	
Publication’s author and publication Year	
Publication’s country	
Types of data used in the articles	
Types of Solution Category the research article proposes	
The algorithm used to create the proposed solution	
Evaluation Metrics used in the proposed model	
Application of PICO in the research article	
The objective of the research article	
The tool used in the research article	

We used a narrative synthesis approach to bring together and summarise the data, grouping and comparing study characteristics to identify commonalities, differences and patterns. The data was categorised into pre-defined categories including AI tool type, study context (e.g. country of origin, healthcare setting), methodology, outcomes (e.g. accuracy, sensitivity, specificity), challenges and limitations. Independent authors reviewed and refined these categories to ensure consistency and alignment with the study objectives. Descriptive summaries and comparative analysis highlighted the key similarities and differences between studies, while summary tables showed study characteristics and main findings.

### Risk of bias and applicability

Risk of bias and applicability were undertaken using the prediction model Risk of Bias Assessment Tool (PROBAST) [21] and Quadas-2 tools [22] for different types of studies. For prediction model evaluation, we have used PROBAST https://www.probast.org/wp-content/uploads/2020/02/PROBAST_20190515.pdf, and for diagnostic assessment, the QUADAS-2 tool https://www.bristol.ac.uk/media-library/sites/quadas/migrated/documents/quadas2.pdf. The first author (SI) applied these tools and the third author (AA) reviewed them to ensure thoroughness and accuracy. To address the issue of inter-rater reliability which is crucial in systematic reviews we used a consensus-based approach. Whenever there were disagreements between the initial assessments of the first and third author these were resolved by the corresponding author (KM).

## Results

Our initial search results included a total of 1743 results which we found using the search query given in Table 2. From these, a sustainable amount of papers were sourced from Science Direct, Wiley, and Springer which consisted of 471, 345, and, 316 respectively. However, only 5 and 3 papers were chosen from Springer and Wiley respectively. On the other hand, 13 papers were chosen from Science Direct finally. Moreover, the largest amount of selected papers came from PubMed and IEEE Xplore which consisted of a total of 242 articles.

**Table 2:** Number of articles based on the database search result and screening

Searched Databases	Search Results	Selected after Title & Abstract Screening	Selected after Full-text Eligibility Check
Science Direct	471	47	13
ACM Digital Library	117	13	4
PubMed	187	37	18
SAGE	252	12	3
IEEE Xplore	55	30	17
Springer	316	22	5
Wiley	345	16	3
**Total**	**1743**	**177**	**63**

It is staggering as 35 of the selected papers out of 63 came from these 2 databases when we see that both had almost the lowest amount of papers in the initial stages. IEEE Xplore had only 55 initial stage papers. The remaining databases were ACM Digital Library and SAGE which contributed only 7 papers which is pretty low considering their initial numbers. As a result, we can see that the paper distribution was pretty random and the quality of the paper did not depend on numbers but rather on the content that the papers represented.

**Fig. 1:**
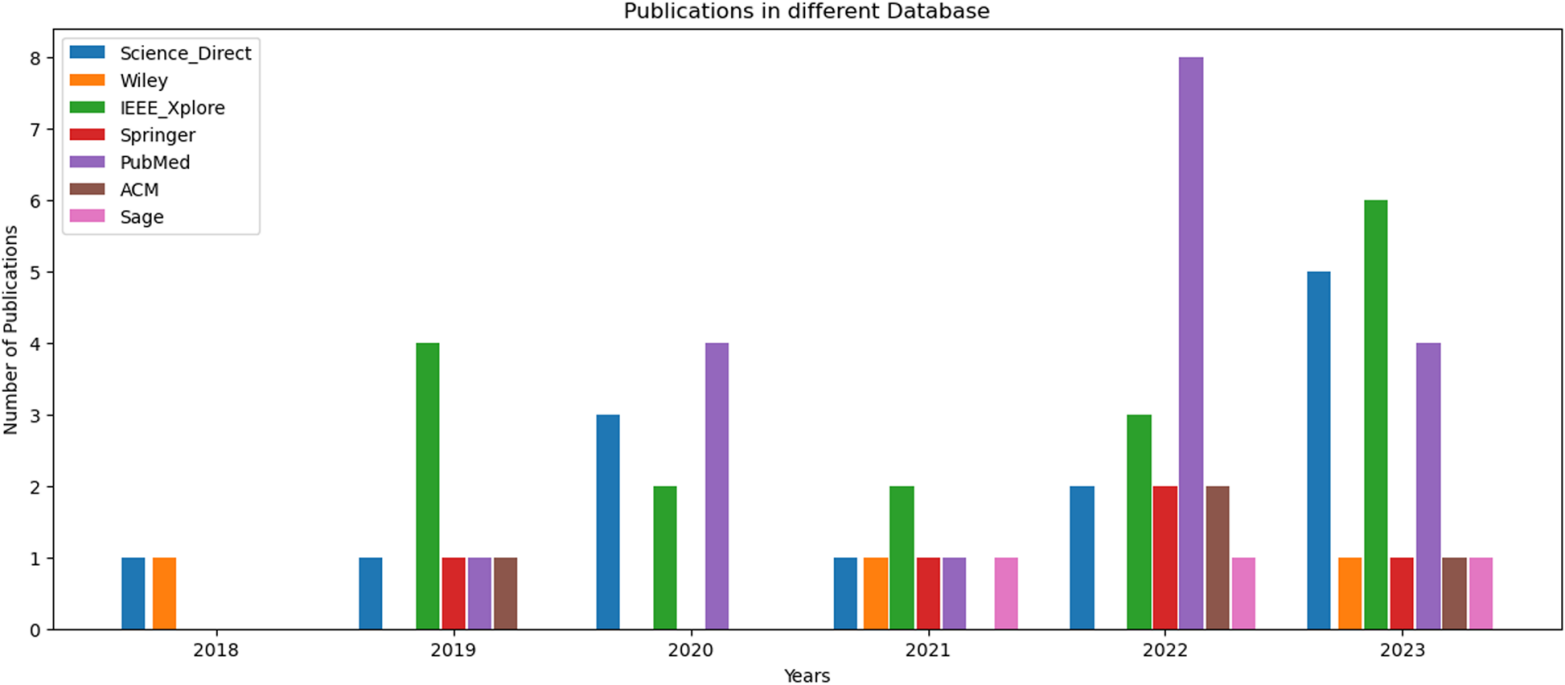
Chart showing the trend in selected research articles published by selected databases (individual database by colour and total by number) from 2018 to 2023

The Fig. 1 illustrates the distribution of papers among different databases from 2018 to 2023. The most productive years were 2022 and 2023, with a combined total of 37 publications, which is more than half of the overall papers.

PubMed had the highest individual year total with 8 publications in 2022. Conversely, only Wiley and Science Direct had publications in 2018, making it the year with the least amount of papers. Additionally, in 2021 there was an almost uniform distribution of papers across databases, except for ACM which had no contribution that year. It is evident that Science Direct consistently published papers each year, closely followed by IEEE Xplore which missed only one publication year. This suggests an incremental trend in paper distribution over these years.

**Fig. 2:**
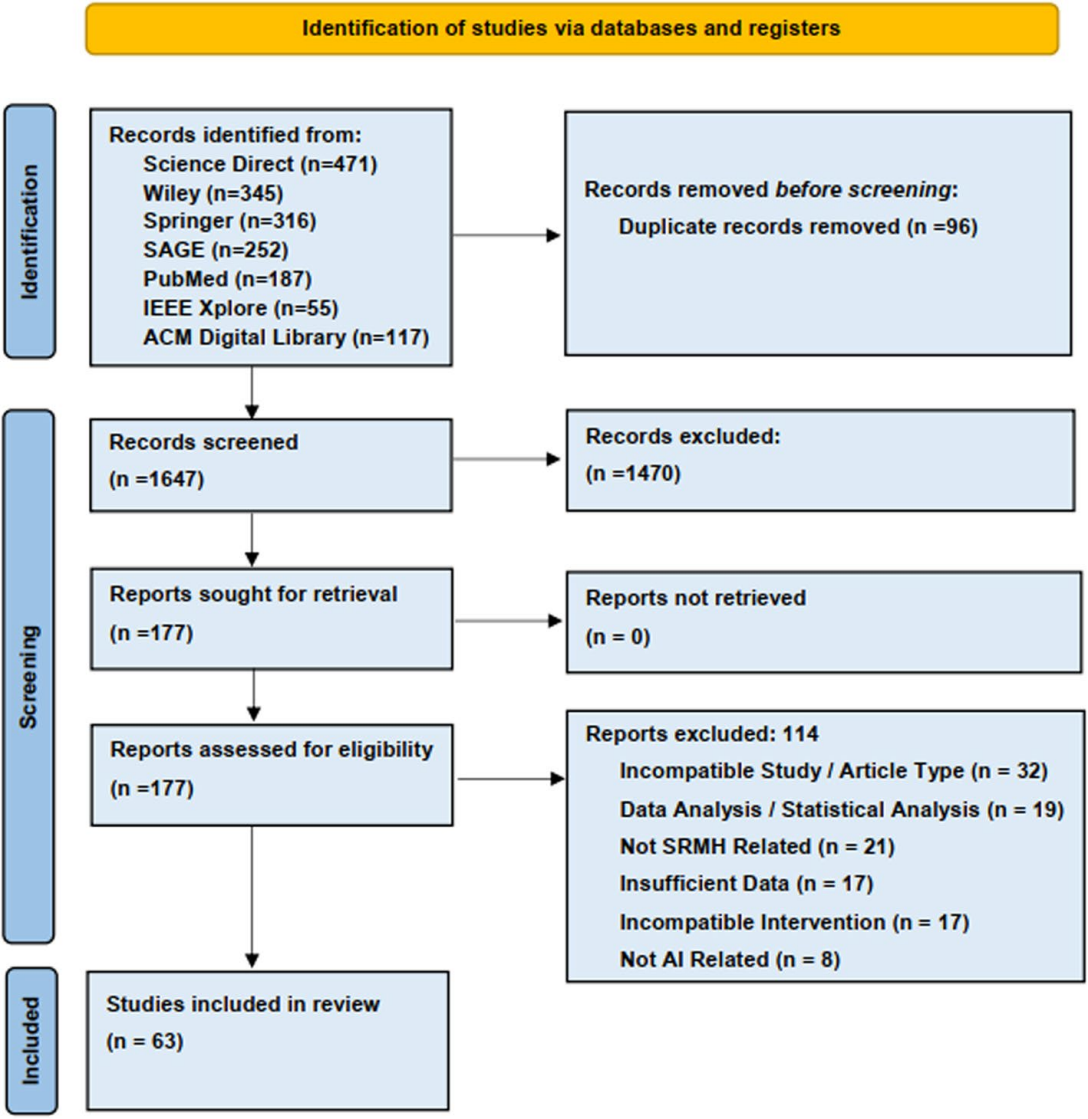
Preferred reporting items for Systematic Reviews and Meta-Analysis (PRISMA) flow diagram showing the process of study selection for this systematic review [20]

The Preferred reporting items summarize the selection procedure for Systematic Reviews and Meta-Analysis (PRISMA) selection flowchart [20] for this study, illustrated in Fig. 2 and the checklist is given in (Additional file 7).

Initially, a total of 1773 papers were collected. After removing duplicates, the number decreased to 1647. Following the first screening, 1470 papers were excluded, leaving 170. Subsequent peer reviews in the second screening identified 63 promising and potentially relevant papers that could be addressed.

In Table 3, we have discussed the characteristics of all 63 articles in the systematic review. The table answers some of the queries related to our research questions. It shows the properties of the following variables which are: Author, publication, Study Design, Purpose, Population, Comparator, Outcome, and Solution Category. As a result, we can gain in-depth knowledge about the different characteristics of our studies and learn how they are interrelated. The extracted data for every article are available in (Additional File 2).

**Table 3:** Characteristics of the studies included in the systematic review

Author	Study Design	Purpose	population	Comparator	Outcome	Solution category
Huang et al 2023 [23]	Retrospective cross-sectional study	Identify major risk factors affecting male sperm count using machine learning predictive models	Male individuals underwent annual health screening	Traditional multiple linear regression	Identified major risk factors affecting male sperm count	Prediction tool
Chen et al 2023 [24]	observational and retrospective.	Develop and evaluate machine learning models for the prediction of cervical cancer	Patients at risk of cervical cancer	Comparison with other machine learning models	performance metrics evaluation for predicting cervical cancer in patients at risk	Prediction tool
Hariprasad et al 2023 [25]	retrospective observational study	Develop an efficient risk prediction model for cervical cancer using machine learning techniques	Female patients	Comparison with existing models	Improved performance metrics in predicting cervical cancer risk	Prediction tool
Al Ghadban et al 2023 [26]	Prospective case-cohort study	Utilize machine learning models on metabolic data to predict spontaneous preterm birth in pregnant women	Pregnant women at risk of spontaneous preterm birth	Standard clinical assessment	Accuracy of predicting spontaneous preterm birth	Prediction tool
Shivangi et al 2023 [27]	observational and analytical	Predict and analyze Polycystic Ovary Syndrome using machine learning techniques	Women with polycystic ovary syndrome	Traditional diagnostic methods	Identification of important indicators	Prediction tool
Allen et al 2023 [28]	Observational study	Utilize natural language processing techniques to indirectly identify psychosocial risks	Pregnant and postpartum women	N/A	Identify psychosocial risks during the perinatal period through the analysis of language patterns	Screening tool
Chen et al 2023 [29]	Retrospective, observational study	Develop and evaluate a machine learning-based prediction model for prostate cancer	Patients with suspected prostate cancer	Comparison with classical PSA predictors	Improved accuracy in predicting prostate cancer diagnosis	Prediction tool
Cersonsky et al 2023 [30]	Observational study	Create and refine machine learning models for predicting stillbirth	Pregnant women	Previous predictive models using logistic regression	Accuracy and sensitivity in predicting stillbirth	Prediction tool
Shen et al 2023 [31]	modelling cost-effectiveness analysis	Assess the cost-effectiveness of AI-assisted liquid-based cytology testing for cervical cancer screening	Women in China	Traditional screening methods	Cost-effectiveness for cervical cancer screening	Screening tool
Reátegui et al 2022 [32]	Observational and comparative study	Identify patterns in sociodemographic and clinical information related to cervical cancer in women with HPV	Women with an active sexual life	Different cytology results, age groups, and marital statuses	Identification of patterns related to cervical cancer	Assessment tool
Xu et al 2022 [33]	Prospective	Develop a web-based risk prediction tool for HIV and STIs using machine learning algorithms	Individuals seeking HIV and STI risk assessment	Traditional risk assessment	Personalized risk assessment for HIV and STIs	prediction tool
Li et al 2022 [34]	Retrospective cohort study	Develop and validate an algorithm for predicting the risk of preeclampsia in pregnant women	Pregnant women	N/A	Improved prediction accuracy and identification of risk factors for preeclampsia	prediction tool
Amitai et al 2023 [35]	Cross-Sectional Study	To predict the risk of first-trimester miscarriage in cleavage-stage embryos during IVF	Women Cleavage-stage embryos	Traditional assessment methods	Prediction of first-trimester miscarriage	prediction tool
Blass et al 2022 [36]	Retrospective cohort	Endometriosis prediction using machine learning algorithms	Women with endometriosis	N/A	Prediction model performance	prediction tool
Pawar et al 2022 [37]	Cross-sectional	Develop a robust machine learning predictive model for maternal health risk assessment	Pregnant women	Traditional machine learning algorithms	Maternal health risk prediction	prediction tool
Xu et al 2022 [38]	Retrospective study	Develop and validate a machine-learning-based risk prediction tool for HIV and three common STIs acquisition over the next 12 months	Individuals at risk of HIV and STI acquisition	N/A	Prediction of HIV and STI acquisition	prediction tool
Jun et al 2022 [39]	Retrospective observational study	Identify the risk factors of cervical cancer in women	Women in a private hospital	Women without cervical cancer	Identification of risk factors for cervical cancer	prediction tool
Bendifallah et al 2022 [40]	Retrospective cross-sectional study	Investigate the potential of machine learning algorithms as a screening approach for patients with endometriosis	Women with endometriosis	Traditional diagnostic methods	Accuracy of endometriosis diagnosis	Screening tool
Nsugbe et al 2022 [41]	Retrospective study	To develop a predictive model for the early detection using machine learning	Women with endometrial cancer or atypical hyperplasia	Standard diagnostic methods	Early and accurate diagnosis of endometrial cancer	Diagnosis tool
Sanderson et al 2020 [42]	Case-control study design	Predicting the risk of death by suicide following an emergency department visit for parasuicide	Individuals visited the emergency department for parasuicide	N/A	Quantification of suicide risk in a clinical setting	Prediction tool
Bao et al 2021 [43]	Retrospective study	Predict the diagnosis of HIV and sexually transmitted infections among men who have sex with men	Men who have sex with men	N/A	Diagnosis of HIV and sexually transmitted infections	Prediction tool
Metsker et al 2020 [44]	Retrospective, observational, data-driven	To develop a data-driven model using machine learning methods for the prediction of a labor due date based on pregnancy history	Female patient with underwent treatment	N/A	Prediction of a labor due date to allow proper resource planning	Prediction tool
Petrozziello et al 2019 [45]	Observational, retrospective cohort study	To develop and evaluate the performance of MCNN for fetal compromise detection	Pregnant women in labor	Traditional visual examination of CTG	Improved accuracy in detecting fetal compromise during labor and delivery	Diagnosis tool
Silva et al 2019 [46]	Experimental	To develop and evaluate the performance of LSTM networks for predicting cervical cancer	Women with suspected cervical cancer	performance comparison of different metaheuristic optimizers	Improved accuracy in cancer diagnosis	Prediction tool
King et al 2018 [47]	Retrospective observational study	To develop and validate triage algorithms to predict STI diagnoses	MSM and young people	N/A	Identification of factors associated with new STI diagnoses	Prediction tool
Bruno et al 2023 [48]	Cross-sectional Cohort study	Explore the potential of machine learning to predict changes in depressive symptoms	Patients with subclinical depression	Symptom changes over time	Forecasting of symptom changes using machine learning	Prediction tool
Foltz et al 2022 [49]	Cross-Sectional Study	Reflect on the nature of measurement in language-based automated assessments of patients’ mental state and cognitive function	Patients undergoing automated assessment	Traditional assessment methods	Improved measurement accuracy and efficiency	Prediction tool
Adeli et al 2023 [50]	Longitudinal study	Dynamically estimate the short-term risk of falls (within the next 4 weeks) using ambient gait monitoring and clinical data	Older adults with dementia	N/A	Estimation of fall risk within the next 4 weeks	Prediction tool
Msosa et al 2023 [51]	Retrospective observational	Develop predictive models for mental health crisis prediction among individuals with depression	Mental health service users	N/A	Development and evaluation of predictive models for mental health crisis prediction	Assessment tool
Ilias et al 2023 [52]	Experimental study	Propose and evaluate a solution for identifying stress and depression in social media using transformer-based models	Social media users	Performance of transformer-based models	Improved model performance to identify differences between stressful and nonstressful texts	Screening tool
Biplob et al 2023 [53]	Predictive study	Use machine learning algorithms to predict suicidal ratios across different continents	Individuals at risk of suicide	Different machine learning algorithms performance	Prediction of suicidal ratio	Prediction tool
Popat et al 2023 [54]	Computational and clinical research study	To explore the use of uncertainty-aware deep learning models for diagnosing mental health conditions using clinical patient health record data	Patients with mental health conditions	Traditional decision-making approaches meaning health care professionals	Improved clinical decision-making	Support tool
Akhlaghi et al 2023 [55]	Prospective observational study	Demonstrate deep Learning models to support clinical decision-making	Patients with mental health conditions	Traditional machine learning models and clinical diagnostic methods	Improved accuracy and reliability of clinical decision-making	Support tool
Berge et al 2023 [56]	Qualitative co-design workshop	Design and evaluate AI-enhanced decision support during telephone triage	Mental health patient	Traditional telephone triage workflow	Improved clinical assessment and documentation	Support tool
Lu et al 2022 [57]	Retrospective cohort study	Develop and evaluate ML models for the prediction of suicidal events	Inmates in a correctional setting	Comparison of the performance of AI algorithms	Improved prediction of suicidal and self-injurious events and enhanced sensitivity and specificity	Prediction tool
Miles et al 2022 [58]	Cohort study	Develop and validate a risk prediction model to support ambulance clinical transport decisions	Conveyed ambulance patients	Standard transport decision-making	Avoidable ED attendances	Prediction tool
Aulia et al 2022 [59]	Experimental study	Develop a machine learning solution for predicting depression	Indonesian Twitter users	Different data scenarios and pre-trained language models	Developed machine learning approaches for predicting depression	Prediction tool
Skaik et al 2022 [60]	Computational research study	Develop and evaluate a predictive model for depression by filling out the Beck’s Depression Inventory questionnaire	Social media users in Canada	N/A	Predicted depression levels	Prediction tool
Monreale et al 2022 [61]	Observational and analytical	Identify mental-health related patient from subreddits	Reddit users with addiction, anxiety, and depression	Performance of different models and subsets of LIWC features	Detection and classification accuracy	Prediction tool
Shea et al 2022 [62]	Observational cohort study	Identify autistic adults enrolled in Medicaid programs	Autistic adults not enrolled in Medicaid	Gold standard clinical assessment	Developed and evaluated a PRE tool to identify Medicaid-enrolled autistic adults	Diagnosis tool
Sun et al 2022 [63]	Mixed study	Improving user control for explainable online symptom checkers	Users of online symptom checkers	Static disclosure of all explanations	Perceived transparency and affective trust	Support tool
McCosker et al 2023 [64]	Qualitative case study	Propose an integrated approach to managing the interaction between human and machine moderators	Mental health patient	Traditional moderation practices	Improved mental health services	Support tool
Rozova et al 2022 [65]	Retrospective analysis	Develop an automated system for detecting self-harm presentations in ED triage notes using NLP	Emergency Department (ED) patients	Traditional machine learning and deep learning Model	Accurate identification of self-harm presentations	Diagnosis tool
Martinez-Eguiluz et al 2023 [66]	Predictive study	To evaluate machine learning algorithms for the classification of Parkinson’s disease	Patients with Parkinson’s disease	Comparison of performance of machine learning algorithms	Prediction of Parkinson’s disease based on non-motor clinical features	Prediction tool
Xu et al 2021 [67]	Secondary anonymous data analysis	suicide detection in online counseling systems	Users aged 11–35	With BiLSTM deep learning model	Identified crisis cases and provided real-time feedback to counselors	Diagnosis tool
Dharma et al 2021 [68]	Computational analysis	Develop a model for predicting mental health disorders among tech workers	Tech workers	Performance of SVM and FCNN algorithms in predicting mental health disorders	Accuracy rate of the algorithms in predicting mental health disorders	Diagnosis tool
Annapureddy et al 2021 [69]	Retrospective observational study	Develop a personalized decision support system to predict the risk of persistent PTSD severity in veterans	Combat veterans suffering from PTSD	Standard care or other predictive models	Improved prediction and prevention of long-term crisis in veterans with PTSD	Assessment tool
Ameratunga et al 2022 [70]	Cross-sectional	To investigate the prevalence and predictors of post-traumatic stress symptoms in injured	Injured New Zealanders	Hospitalised vs. non-hospitalised participants	Prevalence and predictors of post-traumatic stress symptoms	Prediction tool
Simpson et al 2021 [71]	Retrospective cohort study	Evaluate the performance of the (C-SSRS) Screener in predicting suicide risk and self-harm after ED discharge	Adult patients in the emergency department (ED)	N/A	Prediction of suicide risk and ED visits for self-harm	Prediction tool
van der Schyff et al 2023 [72]	Interventional Study	To explore the potential of AI-based chatbots to provide mental health support	Mental health chatbot user	Traditional mental health support	Effectiveness and accessibility	Support tool
Haines-Delmont et al 2020 [73]	Retrospective analysis	Explore the feasibility of using digital phenotyping and machine learning to predict suicide risk using phone measurements	Patients in acute mental health	N/A	Prediction of suicide risk using data collected from the SWiM app	Prediction tool
Lin et al 2020 [74]	Historical cohort study	To utilize machine learning techniques to predict the presence of suicide ideation in military personnel	Military men and women aged 18–50 years	N/A	Prediction of suicide ideation in military personnel based on psychological stress domains	Prediction tool
Baek et al 2020 [75]	Predictive modeling study	Accurately predict the risk of depression based on various contextual factors	Individuals at risk of depression	Traditional regression analysis	Accurate prediction of depression risk based on various factors	Prediction tool
Howard et al 2020 [76]	Predictive modeling study	Benchmark multiple methods of text feature representation and compare their downstream	social media users	N/A	Prediction of risk classification for social media posts	Prediction tool
Ferraro et al 2020 [77]	Predictive and data-driven study	To develop and evaluate automated text classification methods for triaging from online group texts	Internet support groups for mental health	Traditional manual moderation	Identification of crisis posts	Screening tool
Chen et al 2020 [78]	Retrospective observational study	To develop and evaluate natural language processing-based models for predicting short-term emergency department	Adult non-trauma outpatients	Reference model without NLP methods	Improvement in performance metrics (MSE or MAE) for predicting short-term emergency department length of stay	Prediction tool
Si et al 2019 [79]	Cross-sectional study	To distinguish between the speech of patients who suffer from mental disorders causing psychosis	Participant with psychosis	Utilization of word embeddings and CNN	Prediction rate in distinguishing between the speech of psychosis patients and healthy individuals	Prediction tool
Wang et al 2019 [80]	Computational linguistic analysis	To develop a reliable and efficient method for identifying depression risk in Chinese microblogs	Users of Chinese microblogs	N/A	Assessment of depression risk based on microblog content	Assessment tool
Shrestha et al 2019 [81]	Observational study	Address the problem of detecting depressed users in online forums	Users of the ReachOut.com online forum	Comparison of linguistic features to evaluate their effectiveness	Detection of depressed users and identification of at-risk individuals for appropriate support and intervention	Prediction tool
McCoy et al 2019 [82]	Retrospective cohort study	To determine the length of stay in ED	Children and adolescents in ED	Comparison of dimensional psychopathology scores	Length of stay and probability of hospital admission	Prediction tool
Yang et al 2020 [83]	Cross-sectional study	To enhance understanding of the underlying psychosocial factors associated with dementia in older adults to improve diagnosis	Older adults aged 50 years	N/A	Identification of potential risk factors for dementia	Prediction tool
Milne et al 2019 [84]	Retrospective	To evaluate the effectiveness of an automated triage system in improving moderator responsiveness in online peer support	Moderators of online peer support forums	Moderator behaviour before the introduction of the triage system	Evaluation of the accuracy of the triage systems	Support tool
Singh et al 2018 [85]	Observational study	To explore the potential of using mobile phone metadata to automatically assess an individual’s mental health	People with mental health problem	Traditional assessment methods	Automated and accurate mental health assessment	Assessment tool

### Risk of bias within studies

**Fig. 3:**
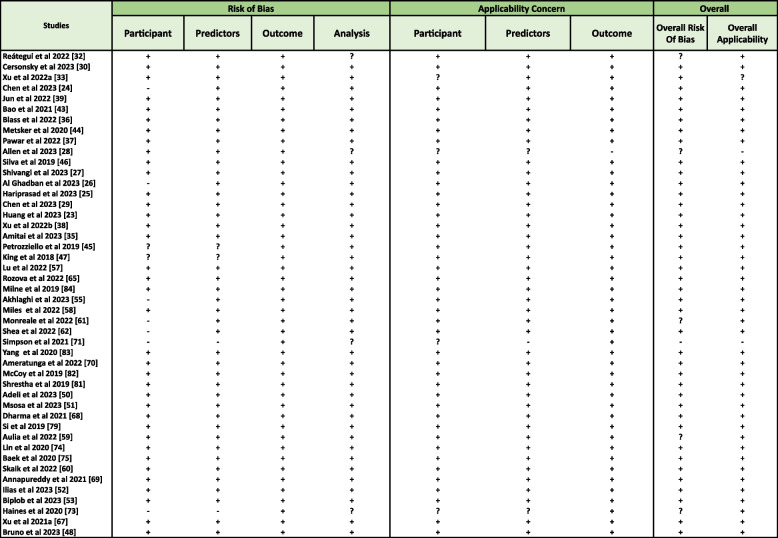
Risk of Bias and Applicability concern summary of PROBAST tool where **’+’** means LOW RISK,**’-’** means High Risk and **’?’** means UNCLEAR Risk

**Fig. 4:**
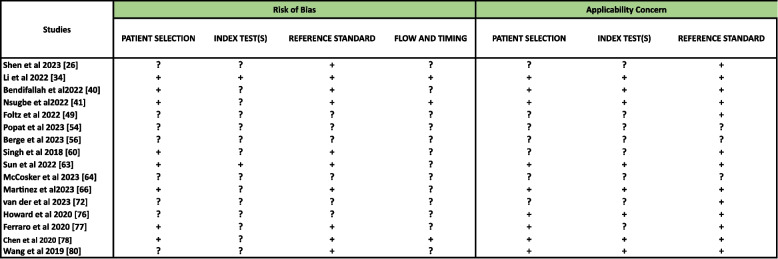
Risk of Bias and Applicability concern summary of QUADAS-2 tool where **’+’** means LOW RISK,**’-’** means High Risk and **’?’** means UNCLEAR Risk

The evaluation of the risk of bias and applicability was conducted using different risk-of-bias tools like PROBAST and QUADAS-2 tools. The results of these assessments are presented in Figs. 3 and 4 respectively, with comprehensive summaries provided in Additional Files 3 and 4.

The PROBAST assessment shows methodological flaws in several studies in Fig. 3, one of which has a high risk of bias [71]. Four studies [29, 44, 58, 59] were classified as unclear risk of bias because of missing data in key areas such as predictor measurement and outcome assessment. The lack of information on managing missing data contributed to the uncertainty around these studies. The other studies had a low risk of bias across most of the PROBAST domains and were more methodologically sound.

The QUADAS-2 tool assessments show the methodological quality across different diagnostic accuracy studies and the risk levels within several domains shown in Fig. 4. Studies like [63] and [41] have mixed risks especially in flow and Timing and lower risks in Reference Standards indicating better methods. The study [34] has low risk across all domains showing good methodological adherence.The study [40] has mixed applicability concerns, especially in the Index Test(s) domain showing potential gaps in the method to real-life clinical practice. Several studies [54, 56, 64] have unclear risks and applicability concerns across all domains showing big methodological gaps. These findings emphasize the need for more methodological transparency and detail to increase the reliability and generalizability of future diagnostic studies and therefore the validity of the methods used.

The search result and the screening stage statistics for the selected databases are illustrated in Table 4. From this analysis, it is evident that the majority of the papers focus on risk prediction in both areas. Out of 63 papers, nearly 70% are dedicated to risk prediction.

**Table 4:** Theme and focus-based categorization of selected research articles in sexual, reproductive and mental health domain

Domain	Theme	Selected research articles	Total
Sexual and Reproductive Health	Triage	[31, 34, 41]	3
	Symptom Checker	[40]	1
	Risk Prediction	[23–30, 32, 33, 35–39, 43–47]	20
Mental Health	Triage	[42, 54–58, 64, 65, 72, 76–78, 84]	13
	Symptom Checker	[49, 56, 61, 63, 72, 85]	6
	Risk Prediction	[48, 50–53, 59–62, 66–71, 73–75, 79–83]	23

Notably, within the mental health domain, out of 25 papers, 22 are centered around risk prediction. Meanwhile, triage is the theme for approximately a quarter (25%) of all papers with a total count of 17. This is followed by a symptom checker which accounts for close to 10% of all papers with a collective amounting to nine publications. Consequently, there appears to be substantial skew and bias towards research related to risk prediction tools in our paper distribution.

In Table 4, the selected research articles were categorized based on their themes where the domain is SRMH and further classified by different tools. The table focuses on providing a comprehensive understanding of the different areas of study within the domain. It was a multi-author effort to ensure a broad and unbiased selection of articles related to AI in risk assessment tools for sexual, reproductive and mental health. This process brought together expertise from across the field of AI in the healthcare field to ensure the data in the table was robust and valid. Each record in Table 4 was evaluated against predetermined criteria and final selections were made by consensus to meet systematic review standards.

As we can see, in Table 4, our domain consists of two parts. which are : i) Sexual and Reproductive Health , ii) Mental Health and these domains are further classified by the risk assessment tools.

### Sexual and reproductive health

Sexual and reproductive health (SRH) plays a significant role in one’s overall welfare, and it is essential to guarantee access to suitable healthcare services and interventions. Utilizing risk assessment tools can enhance the delivery of sexual and reproductive health services by aiding healthcare providers in identifying individuals who may be vulnerable to specific conditions or complications associated with SRH.

**Fig. 5:**
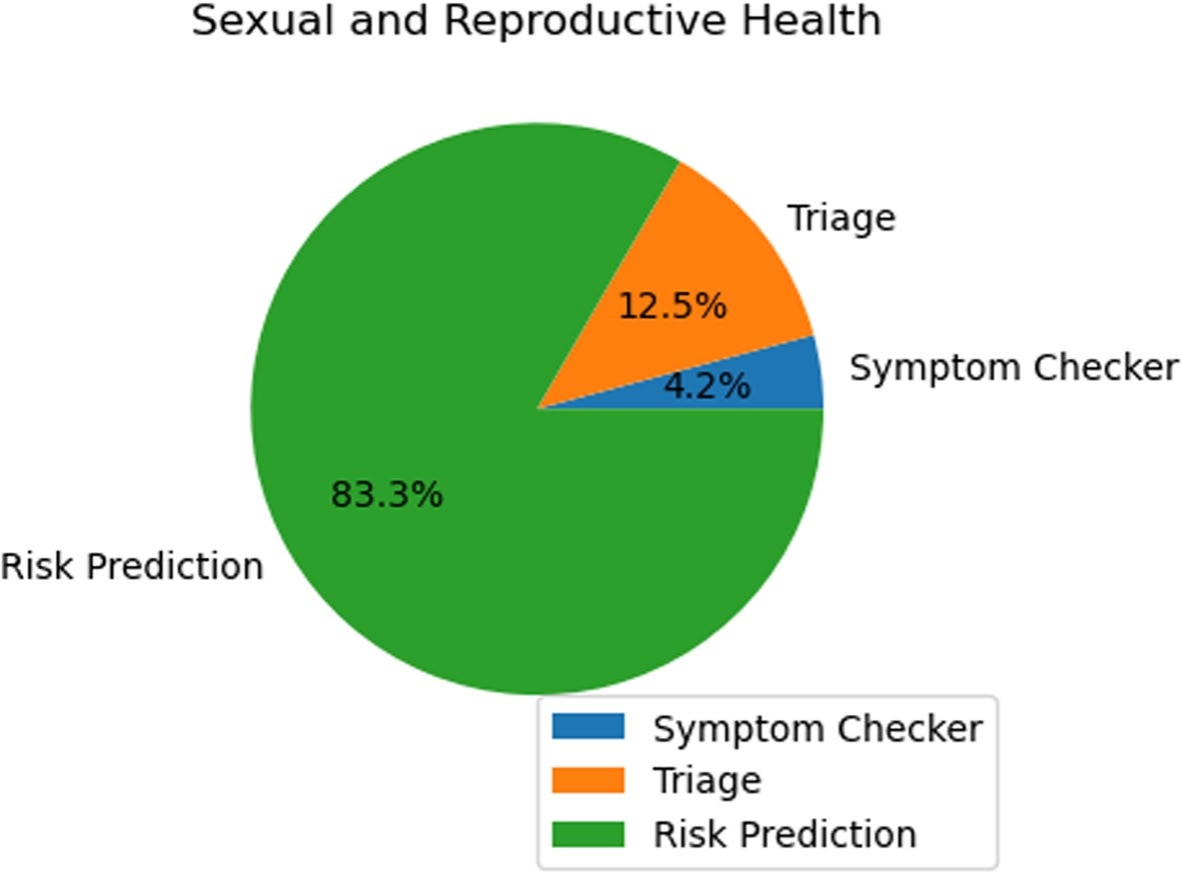
The diagram shows the distribution of risk-assessment tools based articles for the Sexual and Reproductive Health domain

Figure 5 illustrates the distribution of articles related to risk assessment tools within the Sexual and Reproductive Health domain. Notably, the distribution is heavily weighted towards Risk Prediction tools.

#### Triage

Sexual and reproductive health goes beyond the mere absence of illness, encompassing a state of overall physical, mental, and social well-being. Despite being a taboo topic in our society, AI can play a significant role due to its impartiality and anonymity. Endometrial cancer and cervical cancer have substantial effects on women’s health. Studies indicate that analyzing blood spectroscopy triage data can help in identifying endometrial cancer [41]. Also, Artificial intelligence-assisted liquid-based cytology systems have the potential to facilitate the rapid expansion of cervical cancer screening [31]. Moreover, structured EMR data related to triage information might enhance the prediction accuracy for preeclampsia [34]. Various models such as LDA, KNN, SVM, and MLPNN [31, 34, 41] alongside digital phenotyping algorithms have been utilized for screening and predicting diseases like cervical cancer, endometrial cancer, and preeclampsia based on triage notes. Performance evaluations were conducted using metrics such as accuracy, sensitivity specificity, AUC-ROC, F1-score, and PPV/NPV.

#### Symptom checker

Symptom checkers are widely used tools for diagnosing various illnesses related to sexual and reproductive health. They typically involve answering questions about symptoms and medical history to receive a potential diagnosis or recommendation for further medical care. A key feature of these tools is that they are self-supervised, requiring no supervision or control, which makes them unbiased and non-judgmental. These tools can be accessed 24/7, with fast results, and have diverse applications in sexual and reproductive health. Endometriosis is a chronic condition affecting women of childbearing age. It involves inflammatory changes characterized by endometrial-like tissue outside the uterus, estimated to affect 5–10% of reproductive-age women globally. A study focused on developing a comprehensive patient-based screening questionnaire aligned with NHS England guidance on patient involvement in healthcare [40]. This screening tool targets endometriosis based on 16 key clinical and patient-based features, utilizing machine learning algorithms that yield promising results without human intervention. Evidently, screening tools such as symptom checkers have the potential to significantly transform the healthcare industry.

#### Risk prediction

Cervical cancer arises from persistent infection with certain high-risk strains of human papillomavirus and is a leading cause of cancer in women globally. Early detection is crucial for effective treatment. Several research studies have explored various aspects of this disease, including the use of cluster algorithms [32] to detect patterns associated with cervical cancer, identifying risk factors and their correlation with the disease [39], predicting cervical cancer using LSTM models [46], assessing the risk based on lifestyle choices [25], and employing KNN models for detection [24]. Prostate cancer poses a significant threat due to malignant tumor formation in the prostate gland; therefore, efforts have been made to enhance early diagnosis through machine learning-based prediction models as well as visual interpretation techniques [29]. Sexually transmitted infections, which are transmitted through sexual contact, can be effectively addressed by advanced diagnostic methods driven by automation and precision. Among STIs, HIV particularly attacks the immune system. A study has developed a machine-learning-based tool to predict HIV risk as well as the acquisition of three common STIs over 12 months among both males and females [38]. Another study [43] focuses specifically on predicting HIV transmission among men who have sex with other men (MSM) while another [47] explores detection between MSM individuals and young people. Yet another study aims at developing a web-based tool for predicting STI risks using machine learning models [33].

The birth of a healthy child is an essential part of reproductive health. However, pregnancy carries various health risks for women that require careful monitoring and management. Complications such as stillbirth [30], fetal compromise [35], miscarriage [45], and perinatal risks [28] can significantly impact the success of childbirth and contribute to reproductive health issues. Additionally, the prediction of labor duration [44] and preterm birth [26] probability is addressed in specific sources. Hormone disorders like Polycystic Ovarian Syndrome [36] and conditions such as endometriosis [27] pose further challenges to reproductive health, with studies using traditional machine learning models to provide early diagnosis for these diseases. Furthermore, maternal health concerns [37] and male infertility related to sperm count [23] are also predicted through machine learning techniques in particular random forests model, aiming to facilitate early diagnosis efforts towards improving sexual and reproductive health outcomes.

### Mental health

Mental health issues have emerged as a prominent worldwide concern, impacting people from diverse backgrounds and age groups. Within the realm of mental health intervention, the utilization of risk assessment tools is essential for recognizing and appraising potential risks linked to mental health conditions. These tools are formulated to aid healthcare practitioners in gauging the probability of detrimental behaviours like self-harm, suicide, or harm towards others, thereby enabling the deployment of suitable interventions and assistance.

**Fig. 6:**
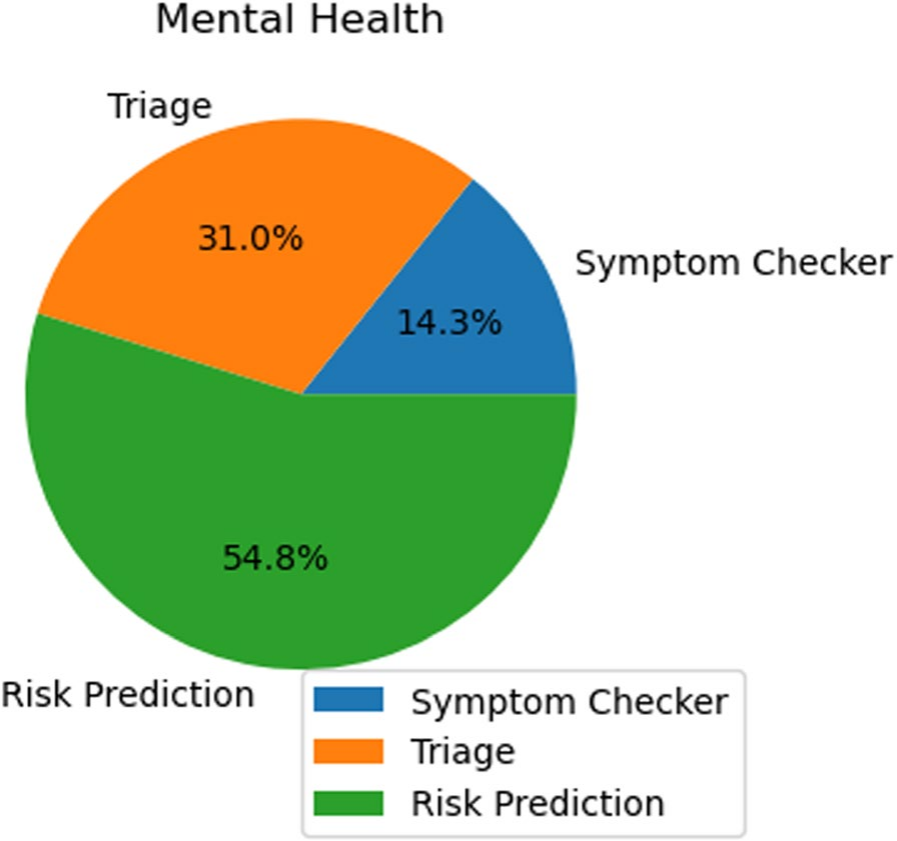
The diagram shows the distribution of risk-assessment tools based articles for Mental Health domain

Figure 6 illustrates the spread of risk-based assessment tools within the mental health sector. Compared to the Sexual and Reproductive Health category, the distribution here appears more balanced.

#### Triage

Mental health triage includes an initial evaluation of individuals with mental illness, usually done over the phone [56] or in person by a mental health professional, aiming to assess the type and urgency of response needed from mental health services or other support systems. Artificial Intelligence has expanded the possibilities for utilizing information, including in the context of mental health triage. It is now feasible to forecast Emergency Department stays [78] and identify suicidal ideation and self-harm through ED Triage [65]. Additionally, events leading to suicide attempts can be better recognized using structured and unstructured data from triage [57]. Triage does not necessarily have to be performed by a clinician; sources such as social media posts [76], online forums [77], and peer support groups [84] can serve as valid triage inputs, creating new avenues for identifying mental health conditions using AI. Furthermore, tailored AI algorithms are designed for specific tasks like predicting admissions, and help make paramedics better transport decisions on scenes that stand to benefit from triage notes [55, 58]. Thus, it becomes apparent that human-machine collaboration plays a crucial role in advancing medical science within the field of mental health [64]. The utilized algorithms exhibited a wide range of methodologies and produced diverse metrics and outcomes, diverging from the traditional approaches. The techniques involved TF-IDF, SVD [78], risk metrics for feature extraction [64], as well as tree-based models like CART and Ensemble [56]. Other papers focused on traditional algorithms, incorporating NLP, SVM, RNN, BERT, Xgboost [55, 57, 65, 77] are used for prediction from triage notes. There were some exceptions such as the Leora model, transfer learning, ML-based triage, Monte Carlo Dropout, and Variational Inference found in research articles [54, 72, 76, 84]. Various performance evaluation metrics were employed, including MSE, MAE, AUC-ROC, F1-score, and Recall. Additionally, less conventional measures like urgency, similarity score, and prioritization of work by risk were also used. Surprisingly, the GAD-7 scale (Generalized Anxiety Disorder 7-item scale) and PHQ-9 scale (Patient Health Questionnaire 9-item scale) were also incorporated to obtain clinician or expert opinions [72]. The conventional method involves consulting a specialist whose viewpoints may lack precision. Conversely, AI models rely on data from triage or other sources and are programmed to strive for maximum accuracy. This decreases the sole reliance on human opinions and emphasizes the significant impact of AI-based models.

#### Symptom checker

The increasing demand for online healthcare and recent advances in artificial intelligence have sparked interest in automated health tools such as online symptom checkers. Several research papers have utilized symptom checkers as a tool alongside AI. In a nurse-AI collaboration setting, the use of symptom checkers was explored with discussions on enhancing their capabilities to address complex and trivial real-life scenarios [56]. These platforms can also leverage the metadata of phones [85] and posts from Reddit [61] to extract symptoms and identify diseases related to mental health, functioning similarly to traditional symptom checkers. Additionally, AI has shown potential in revolutionizing the health sector through chatbots that can serve as symptom checkers, aiding in the identification of mental health problems while offering 24/7 accessibility without promoting stigma [72]. However, there are concerns about transparency and user-centricity with these mediums which may lead to distrust and misinterpretation [63]. The application of language-based assessment using similar online symptom-checking mediums reflects an innovative approach toward evaluating patients’ mental state and cognitive function [49]. Furthermore, these innovative tools and approaches demonstrate the potential of AI in improving the accessibility, accuracy, and efficiency of mental health care. For mental health concerns, these tools can be extremely beneficial as they can offer self-led early diagnosis leading to efficient management of both time and resources.

#### Risk prediction

Approximately 703,000 individuals worldwide die by suicide annually, accounting for over 1.3% of all deaths in 2019. Suicide and suicidal thoughts are significant contributors to mental health-related fatalities. Identifying the risk of suicide can be a crucial advancement that may help prevent premature loss of life. Following discharge from an emergency department after a suicide attempt leads to an increase in subsequent suicidal behaviours [71]. A study assesses the effectiveness of the C-SSRS screener in predicting self-harm and suicide risk post-discharge from the emergency department. Additionally, various studies have explored methods for predicting suicide attempts using data collected from electronic devices [73] such as apps or algorithms utilizing counselling information [67]. The domain knowledge-aware risk assessment model is one algorithm utilized for identifying potential suicides within online counselling systems. Furthermore, research has been conducted on military personnel who commit suicide due to stress and other psychological factors [74]. It is also plausible to create a model that forecasts variations in the rate of suicides across different continents [53].

Anxiety and depression are common problems that disrupt mental health. These issues are challenging to quantify due to various contributing factors, but several studies have been conducted to address them. Research has validated psychometric profiles [61], detected depressed users through online forums [81], predicted crisis patients with depression using electronic health records (EHRs) [51], assessed Chinese micro-blogs [80] and evaluated Twitter user profiles in Indonesia [59]. Other studies have focused on automatically filling out Beck’s Depression Inventory Questionnaire in Canada [60] and using social media to filter out depressive thoughts [52]. Additionally, research has forecasted symptom changes among sub-clinical depression patients over time [48] as well as focused on predicting the risk of depression using context-based deep neural network models via multiple regression analysis [75]. We also see a discussion about services and support for autistic adults in [62], dementia prediction [83] including whether the patient will fall within two weeks in a cost-effective manner [50]. Predictors of post-traumatic stress disorder and PTSD prediction efforts concerning veterans’ precursors are detailed in references [70] and [69].

AI-based risk assessment tools in sexual, reproductive and mental health (SRMH) are game changers especially in low to middle-income countries (LMICs). These tools improve diagnostic accuracy, machine learning models are more sensitive and specific in detecting mental health conditions than traditional methods. For example, AI-based systems have shown better prediction of psychotic disorders and can intervene earlier. In reproductive health, AI-based models can predict high-risk pregnancies like preterm births and can trigger clinical actions that reduce neonatal mortality. Symptom checkers powered by AI chatbots can do self-assessment for STIs and promote earlier health-seeking behaviour in low-resource settings. These examples show how AI can improve predictive accuracy, early detection access to healthcare and equity in SRMH.

## Discussion

In our comprehensive literature review, we assessed the application of artificial intelligence in tools such as triage, symptom checkers, and risk prediction. Upon examining the included studies, a clear pattern emerges concerning the number of publications over time.

**Fig. 7:**
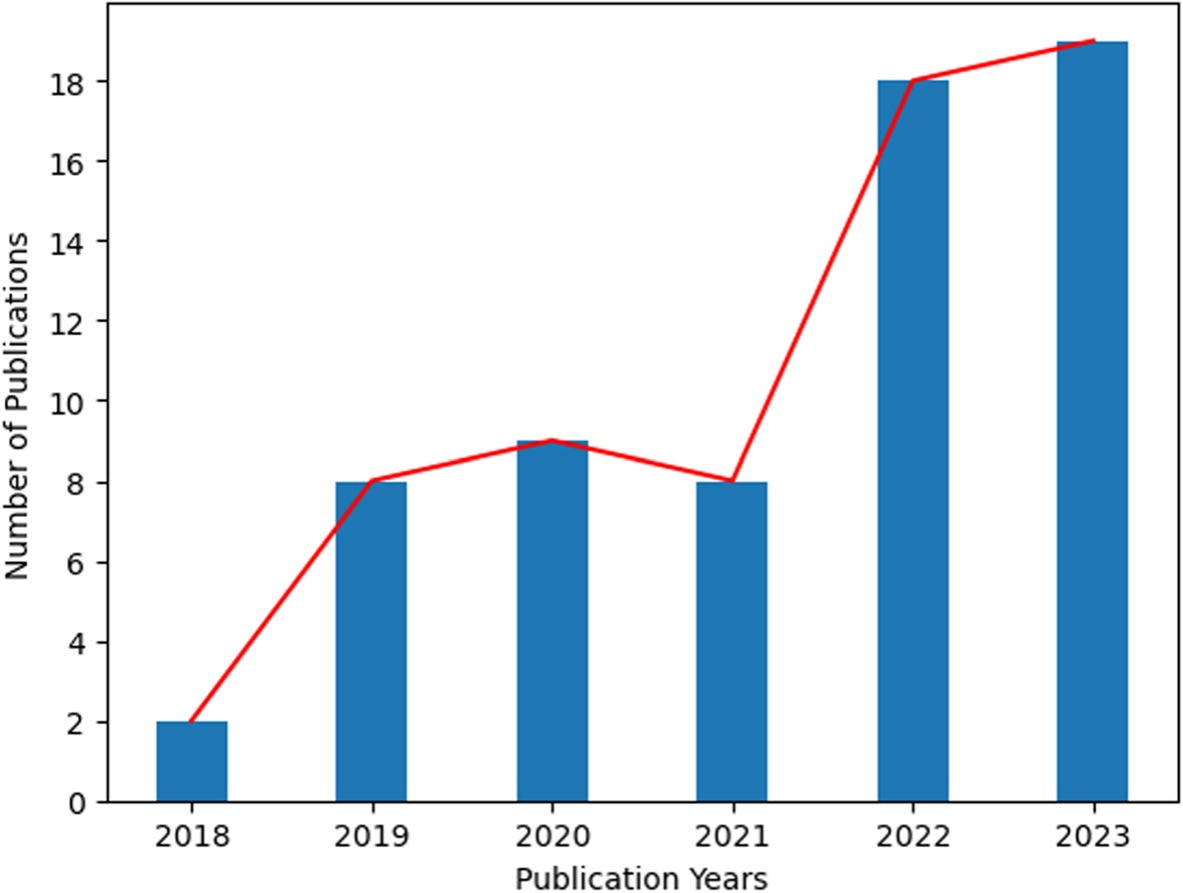
Trends of 63 selected articles according to publishing year. The red trend line is going upwards which represents the research on risk assessment tools on Sexual, Reproductive, and Mental Health is increasing

In Fig. 7, we can see a discernible upward trend in publication numbers as the year progresses, with 2023 marking a peak year featuring 19 publications. This indicates a consistent increase in publications over time, with only 2021 standing out as an exception.

**Fig. 8:**
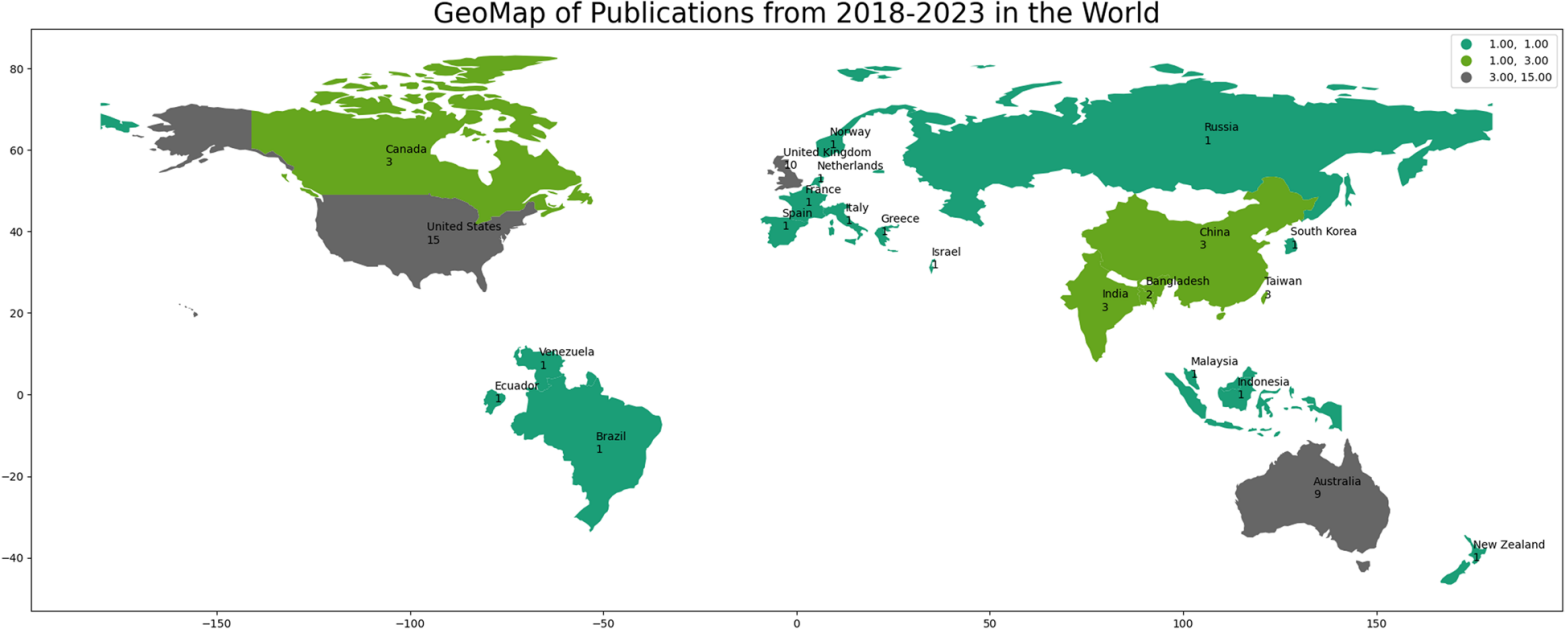
This geographic map shows 63 research articles from different global locations. Grey represents regions with a higher number of publications and green represents areas with fewer articles published

In Fig. 8, the geographical map illustrates the distribution of publications across 23 countries. Variations in publication frequency can be easily identified through color and intensity differences on the map. The colors on the map indicate the distribution of published articles in each region, with grey representing regions with a higher number of publications and green representing areas with fewer articles published. The United States (USA), United Kingdom (UK), and Australia are at the forefront with 15, 10, and 9 publications, accounting for more than half of the total publications. This map indicates a high volume of publications from developed countries. Additionally, Asian nations such as China and India also have notable publication numbers, followed by Bangladesh and other countries. These findings highlight the significant impact of limited research activities in developing countries. The statistics reflect this same result.

**Fig. 9:**
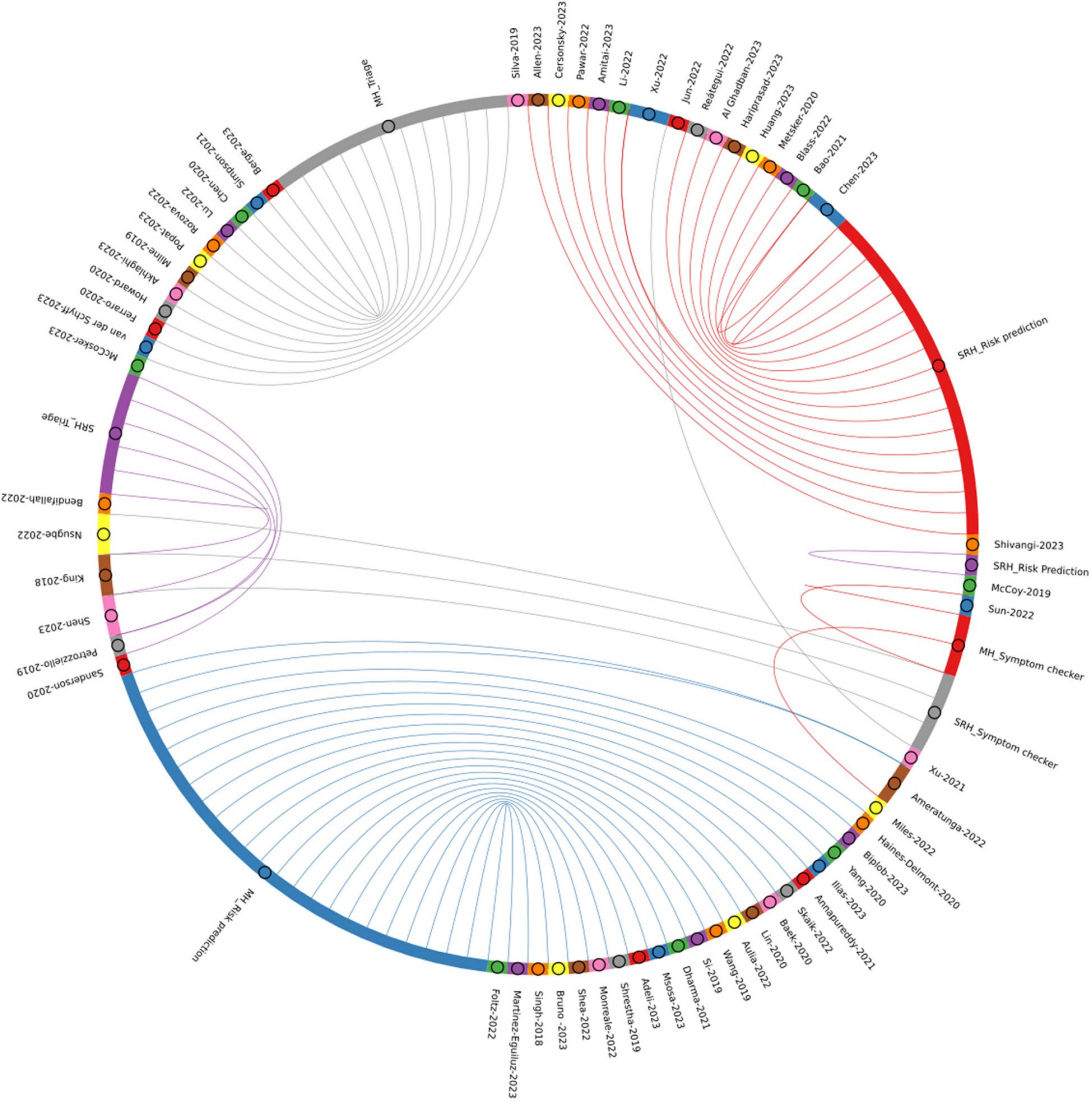
Chord Diagram depicting risk assessment tools for Sexual, Reproductive and Mental Health as documented in systematic review papers. The visualization illustrates the connections between the assessment tools and the articles

In Fig. 9, the chord diagram shows the relationship between different tools based on sexual, reproductive health, and mental health with the selected articles. Here, individual tools are associated with specific colors. The tools are triage, symptom checker, and risk prediction and are further classified by sexual, reproductive, and mental health.

In the last 5 years, there has been an increase in the number of tools that have utilized already existing methods in statistics and computer science. This may be due to the widespread availability of data worldwide. This systematic review identified 63 studies that aimed to distinguish the use of AI in different risk assessment models. Our paper is based on the use of AI in different risk assessment tools for SRMH. We found a dominance of risk prediction tools in both Sexual and Reproductive Health, and Mental Health. This disparity is caused because of the pragmatics of AI. As there are numerous feature variables, risk prediction tools are used more than symptom checkers and triage-based tools.

We can see that symptom checkers are used in a very low number of papers. The reason could be that there is widespread use of AI. As a result, methods that require self-supervision are becoming less used as data is getting more abundant, and the risk prediction tools that are used can guarantee privacy, which is the main reason for using symptom checkers that offer anonymity. Also, triage is not used that frequently, which shows that triage has not yet become a dominant factor in risk assessment. This can be due to the fact that NLP for feature extraction is still developing. Also, triage is not used in every medical scenario.

The evidence from the studies demonstrates the evident incorporation of AI in healthcare accessibility, decision support systems, monitoring, and tracking. Risk prediction tools are the most commonly used tools, which make prediction models and classification models the most prevalent.

The included papers encompass a wide range of data types and sources collected from various studies across multiple disciplines. These include, among others, health screening indicators data obtained from MJ Group, a well-known health screening centre in Taiwan, and healthcare records sourced from the Hospital Universitario de Caracas in Venezuela. Additionally, valuable insights come from cervical cancer risk factor data available in the UCI repository and metabolomic data derived from participants in the Pregnancy Outcome Prediction study at the Rosie Hospital. Electronic health record data from Mersey Care in the UK and suicidal ratio data from the Kaggle platform add significant dimensions to the review. Furthermore, social media posts extracted from Indonesian and English text on platforms such as Twitter and Reddit provide unique perspectives. Other included sources encompass clinical consultation data, smartphone-collected user-generated and clinical information, and forum post data from platforms like ReachOut.com. This comprehensive collection of diverse datasets not only enhances the systematic review but also facilitates a subtle understanding and synthesis of findings across a broad range of research areas.

The most used algorithms in the selected articles (Additional file 6) respectively Random Forest (RF), Logistic Regression (LR), and Support Vector Machine (SVM) are known for their versatility in classification and regression tasks. While K-nearest neighbours (KNN) and Decision Trees (DT) are widely used because of their ease of interpretation and implementation, Linear Regression is still a basic method for modelling connections between variables also used in a few articles. Deep learning algorithms were used for their wide use in applications such as Neural Networks (NN), CNN, and LSTM. Also, NLP was used widely in the application of triage. A Large Language Model (LLM) namely BERT is only used in a few articles [26, 53, 59, 68, 72, 75, 76] for triage-related services and others. The use of other LLM models in risk assessment services is very low in number. Less frequently mentioned algorithms, including DBSCAN, Markov Models, Probit Regression (PR), CART, Leora Model, and Poisson Models, highlight specialized applications in particular fields.

The choice of evaluation metric depended on the type of intervention used, and as the majority of them were risk prediction tools, metrics such as accuracy, precision, recall, F1-score, and specificity were widely used. These metrics focus on classification performance, balancing true positives, false negatives, and false positives. Other metrics include the Correlation Coefficient, Mean Squared Error, Gower distance, Bayesian statistical tests, incremental cost-effectiveness ratios, C-index, and Negative Predictive Value. These metrics emphasize the model’s discriminatory ability and performance in negative classes. The (Additional File 5) shows the whole overview of the used evaluation metrics in the respective articles.

We connect our findings to practical implications, showing how AI can impact SRMH. Our review shows that AI technologies can democratize health access through advanced diagnostics and personalized interventions, for underserved populations. For example, AI models can detect early and tailored plans that fit individual health profiles, reducing health outcome disparities. But our analysis also warns against the unintended reinforcement of existing inequalities through these technologies. For instance, biases in AI algorithms, from non-representative training data, can lead to health outcomes that disproportionately affect marginalized groups. And disparities in access to digital technology can widen the gap between socio-economic groups. To address these, we propose targeted interventions like guidelines for inclusive AI training and policies to ensure equitable access to AI-based health services. By linking our findings to action we want to give a more detailed view of how AI can bridge or widen health disparities, so future research and policy can be more equitable health solutions.

AI-based tools such as triage systems, symptom checkers, and risk prediction models have the potential to significantly enhance healthcare delivery, particularly in areas where resources are limited. However, the extent of this impact is contingent upon overcoming current challenges related to data diversity, ethical considerations, and system integration.

To assess the performance of the AI tools a thorough comparison between the tools and traditional methods needs to be done. For diagnostic accuracy, the performance of machine learning models like CNNs in medical imaging to the subjective assessment of radiologists will be compared. Treatment personalization will be explored by comparing how AI can tailor cancer therapies based on genetic profiles versus traditional methods. Operational efficiency will be looked at by comparing AI-driven scheduling and data management to manual, error-prone traditional methods and showing the potential for resource optimization. Patient monitoring will compare AI’s ability to collect real-time, continuous data via wearables to traditional methods of periodic checkups and show AI’s role in timely interventions. We will also discuss the ethical and privacy implications to contrast the challenges of AI. These are not all, but only some applications of AI that are being used over traditional methods.

However, AI tools also have limitations that must be acknowledged. AI models are complex and can be “black boxes” where the decision-making process is not transparent and practitioners can’t fully trust or understand the basis of the AI decision. Integrating AI into existing healthcare systems is a big challenge requiring significant changes to current workflows and systems which can be resource-intensive and slow to implement. These limitations show that AI-based tools having far-reaching potential are still problematic to adapt directly in healthcare and as a result traditional tools are still a viable option to rely on.

In our systematic review of AI applications in sexual, reproductive and mental health (SRMH) particularly in low- and middle-income countries (LMICs) we looked at the ethical considerations of the included studies, particularly informed consent, transparency and accountability. Although we didn’t collect data or interact with patients so didn’t need informed consent for our own research, we evaluated how each study addressed ethical issues. This included looking at their ethical approval process and how they managed consent, transparency and accountability in their research framework. The reviewed papers [26, 33, 37, 54, 55, 58, 64, 72, 73, 84] demonstrate a strong commitment to ethical considerations and informed consent in healthcare research, particularly in sensitive areas such as mental health and cancer detection. Most studies obtained ethical approval from relevant institutional review boards, ensuring adherence to established guidelines that protect participant rights. Informed consent was explicitly secured from participants in many studies, fostering transparency and trust between researchers and subjects. Some studies appropriately sought waivers for informed consent in retrospective research, emphasizing minimal risk to participants. Overall, these practices reflect a dedication to ethical integrity and accountability, which are essential for fostering trust in the research process and ensuring the ethical application of findings in clinical settings.

As noted in Fig. 8, there has been a growing disparity in the quality of published articles between the developing and developed countries. Addressing this disparity demands, cooperation and open-access initiatives involving global institutions which may contribute to more accessible AI applications in particular domains, as well as testing of ways established research entities could improve the capability for research locally within underdeveloped nations. Adherence to these principles may lead to a much wider availability of varied and comprehensive data sets, with global benefit for researchers. These datasets can cover a variety of cultural, ethnic, and regional views so that the AI models created may produce highly accurate results without running into societal context issues.

### Combined gap analysis

Numerous research studies have explored various aspects of health and medical research. From a clinical practice perspective, significant gaps still exist that limit the general applicability and robustness of the findings. Many studies have relied on small and uniform datasets from single locations which restricts their relevance to wider and more diverse populations. The frequent use of retrospective designs and self-reported data can introduce biases and inaccuracies. Some studies also overlook important factors such as genetic influences, clinical impacts, and specific demographic characteristics that could significantly impact outcomes. Methodological constraints include choosing machine learning algorithms without adequate validation, underscoring the necessity for evidence-based approaches in clinical practice. There are existing limitations in machine learning applications due to imbalanced data leading to overfitting when applied across different populations. Additionally, Gaps exist in comparing proposed models with traditional methods which limits understanding of relative effectiveness. The use of the Large Language model (LLM) is limited in this type of AI-based health risk assessment tool. Furthermore, Insufficient feature selection descriptions affect reproducibility. Ethical concerns such as ensuring data privacy and ensuring transparency in AI models have remained underexplored. There is an evident deficiency in considering the potential computational resources required for implementing advanced models along with addressing patient data privacy issues to ensure patient safety and trust with AI applications.

### Limitations

We acknowledge several limitations that might impact the generalizability and robustness of our results. We reviewed studies from 7 databases from 2018 to 2023. However, we recognise that our focus on English language articles from high-impact sources might not capture regional differences or local studies, and might miss important variations and insights. This limitation, along with the time frame and databases we used, might have excluded studies outside the scope of our review and therefore limited the depth and breadth of our findings. The search was further limited by the specific search terms we used and we might have missed studies that used different terminology. Not all included studies were of the same quality and we couldn’t do a meta-analysis as the methodological details were not uniform and made data extraction or analysis impossible. We excluded book chapters, conference abstracts, unpublished reports, non-English articles and other grey literature and this might have resulted in an incomplete representation of the evidence and therefore limited our search strategy and the conclusions we drew. Some of the studies in our review had small datasets which might result in overfitting bias and the results might not be applicable to larger populations. Cases of over-representation with imputed or incomplete data might be invalid or unreliable and we need to interpret our findings with caution and not apply them in real-life scenarios.

### Future research direction

In order to enhance future research on AI-based risk assessment tools in sexual, reproductive, and mental health, we must prioritize the creation of extensive, diverse datasets that accurately reflect the broad spectrum of population characteristics, including those of underrepresented groups. This is key to minimising bias and generalisability of AI models. We need to refine diagnostic and intervention strategies for different populations and their unique health challenges. This means establishing standardised performance metrics for the clinical implementation of predictive models so they can be used in real-time crisis management. Integration with national databases and interhospital systems is critical as it will feed the models with comprehensive patient data and facilitate collaboration between healthcare providers and researchers. We need to address ethical considerations, especially in low-middle-income countries where access to healthcare is limited and disparities are huge. Moreover, advancing AI methods like federated learning and deep learning will improve the accuracy and ethical deployment of these tools and build trust and adoption in healthcare. Using Large Language Models (LLMs) for explainability will improve collaboration with healthcare providers and therefore treatment outcomes and operational efficiency. These are the building blocks to tackle the complexities of AI in healthcare and set the stage for big progress.

## Conclusion

In this review, 63 articles were analyzed to look at the use of AI in various risk assessment tools for sexual, reproductive and mental health from 2018 to 2023. The analysis showed a majority of risk prediction tools and significant biases and disparities in the models and datasets used. Our findings suggest global and interdisciplinary collaboration is needed to address the issues identified. Future research should focus on increasing the diversity and inclusivity of datasets to mitigate biases and ensure fair AI use. Robust methods are needed to generalise AI models across different populations and healthcare settings. Interdisciplinary collaboration will be key to achieving this and international frameworks to promote ethical standards and transparency in AI research and deployment. We acknowledge limitations in this review as we excluded non-English studies and only used published literature which may introduce publication bias and limit perspectives. Addressing these gaps by including multi-language research and broader literature bases will be essential to developing fair and effective AI tools and making a big impact on global health. This holistic approach will ensure we get the most out of AI not just in SRMH but in global health too.

## Supplementary Information

Additional file 1. Advanced search string.

Additional file 2. Article Characteristics.

Additional file 3. Included studies Risk of Bias.

Additional file 4. Included studies Risk of Bias.

Additional file 5. Algorithm checklist.

Additional file 6. Matrics checklist.

Additional file 7. Preferred Reporting Items for Systematic Reviews and Meta-Analyseschecklist.

## Data Availability

No datasets were generated or analysed during the current study.
