# Supplementary material for: Artificial intelligence-based risk assessment tools for sexual, reproductive and mental health: a systematic review
Source: BMC Med Inform Decis Mak. 2025 Mar 17;25:132. doi: 10.1186/s12911-025-02864-5 (PMC11912754; doi:10.1186/s12911-025-02864-5)
Supplement: Supplementary file 1 — Additional file 1. Advanced search string. [file 12911_2025_2864_MOESM1_ESM.docx]

| **Database** | **Search Query** |
| --- | --- |
| **Science Direct** | (“Artificial Intelligence” OR “Machine Learning” OR “Natural language Processing” OR “Large Language Model”) AND (“Triage” OR “Health Risk prediction” OR "Symptom checker") AND (“Sexual Health and Reproductive health” OR “Mental health”) |
| **PubMed** | ((("Artificial intelligence" OR "Machine Learning" OR "Natural language Processing" OR "Large Language Model")) AND (("Triage" OR "Health Risk prediction" OR "Symptom checker"))) AND (("Sexual Health" OR "Reproductive health" OR "Sexual and Reproductive Health" OR "Mental health")) |
| **SAGE** | ("Artificial intelligence" OR "Machine Learning" OR "Natural Language Processing" OR "Large Language Model") AND ("Triage" OR "Health Risk prediction" OR "Symptom checker") AND ("Sexual Health" OR "Reproductive Health" OR "Sexual and reproductive health" OR “Mental health") |
| **ACM Digital Library** | [[All: "artificial intelligence"] OR [All: "machine learning"] OR [All: "natural language processing"] OR [All: "large language model"]] AND [[All: "triage"] OR [All: "health risk prediction"] OR [All: "symptom checker"]] AND [[All: "sexual and reproductive health"] OR [All: "mental health"]] |
| **Springer** | (“Artificial intelligence” OR “Machine Learning” OR “Natural language Processing” OR “Large Language Model”) AND (“Triage” OR “Health Risk prediction” OR "Symptom checker") AND ("Sexual Health" OR "Reproductive Health" OR “Sexual and reproductive health” OR “Mental health”) |
| **IEEE Xplore** | (“Artificial intelligence” OR “Machine Learning” OR “Natural language Processing” OR “Large Language Model”) AND (“Triage” OR “Health Risk prediction” OR "Symptom Checker") AND ("Sexual Health" OR "Reproductive Health" OR "Sexual and reproductive health" OR "Mental Health") |
| **Willey** | (“Artificial intelligence” OR “Machine Learning” OR “Natural language Processing” OR “Large Language Model”) AND (“Triage” OR “Health Risk prediction” OR "Symptom checker") AND ("Sexual Health" OR "Reproductive Health" OR “Sexual and reproductive health” OR “Mental health”) |

**Table: Advance Search Query for Different Database**

**Key Terms:** “Artificial Intelligence”, “Triage”, “Symptom Checker”, “Risk Prediction”, “Sexual and Reproductive Health”, “Mental Health”.
